# Strategies for labelling of exogenous and endogenous extracellular vesicles and their application for in vitro and in vivo functional studies

**DOI:** 10.1186/s12964-024-01548-3

**Published:** 2024-03-09

**Authors:** Marie Boudna, Andres Delgado Campos, Petra Vychytilova-Faltejskova, Tana Machackova, Ondrej Slaby, Kamila Souckova

**Affiliations:** 1 Central European Institute of Technology, Masaryk University, Kamenice 753/5, 625 00 Brno, Czech Republic; 2 Department of Biology, Faculty of Medicine, Masaryk University, Kamenice 753/5, 625 00 Brno, Czech Republic

**Keywords:** Extracellular vesicles, Exosomes, EV tracking, Fluorescent labelling, Bioluminescent labelling, Radiolabelling, Magnetic resonance, In vivo imaging, Cre-loxP, CRISPR-Cas

## Abstract

This review presents a comprehensive overview of labelling strategies for endogenous and exogenous extracellular vesicles, that can be utilised both in vitro and in vivo. It covers a broad spectrum of approaches, including fluorescent and bioluminescent labelling, and provides an analysis of their applications, strengths, and limitations. Furthermore, this article presents techniques that use radioactive tracers and contrast agents with the ability to track EVs both spatially and temporally. Emphasis is also placed on endogenous labelling mechanisms, represented by Cre-lox and CRISPR-Cas systems, which are powerful and flexible tools for real-time EV monitoring or tracking their fate in target cells. By summarizing the latest developments across these diverse labelling techniques, this review provides researchers with a reference to select the most appropriate labelling method for their EV based research.

## Background

Extracellular vesicles (EVs) are membranous structures secreted by cells, and depending on their biogenesis, they comprise two main subtypes: exosomes and ectosomes. Exosomes originate from intraluminal vesicles that are formed by the inward budding of endosomal membranes during the formation of multi-vesicular endosomes (MVE), and are released to the cell exterior upon the fusion of MVE with the plasma membrane. Ectosomes are generated at the plasma membrane from its outward budding, followed by membrane fission and their subsequent release from the cell surface [1].

Apart from their biogenesis pathway, which might be difficult to determine, EVs can be characterised by their physical characteristics such as size or density, biochemical composition, or by their cell of origin. Regarding their size, small EVs are generally regarded as membranous vesicles with a diameter of up to 200 nm, as opposed to medium/large EVs exceeding this suggested size range [2]. For the assessment of EVs by protein composition, the MISEV2023 guidelines recommend investigating proteins from two categories that evaluate the presence of EV features. These include proteins associated with the cell plasma or endosomal membrane, such as CD63, CD81, CD9, and cytosolic proteins that are recovered in EVs (e.g. ALIX, TSG101, syntenin). Importantly, the guidelines do not recommend any specific molecular markers for distinguishing between EV subtypes, highlighting the current understanding that no single set of protein markers can definitively categorize EV subpopulations [3, 4].

Aside from the general EV characterization, there is a considerable effort to analyse their active cargo content and determine the EV function under physiological or pathological states. Although they were initially considered only cell waste carriers [5], numerous studies have demonstrated that cells actively release EVs to mediate cell-to-cell communication at adjacent or distant sites [6–10]. Information is transferred in the form of nucleic acids, lipids, and proteins and can influence the physiological state of the recipient cell after the EV content is taken up [11].

**Fig. 1:**
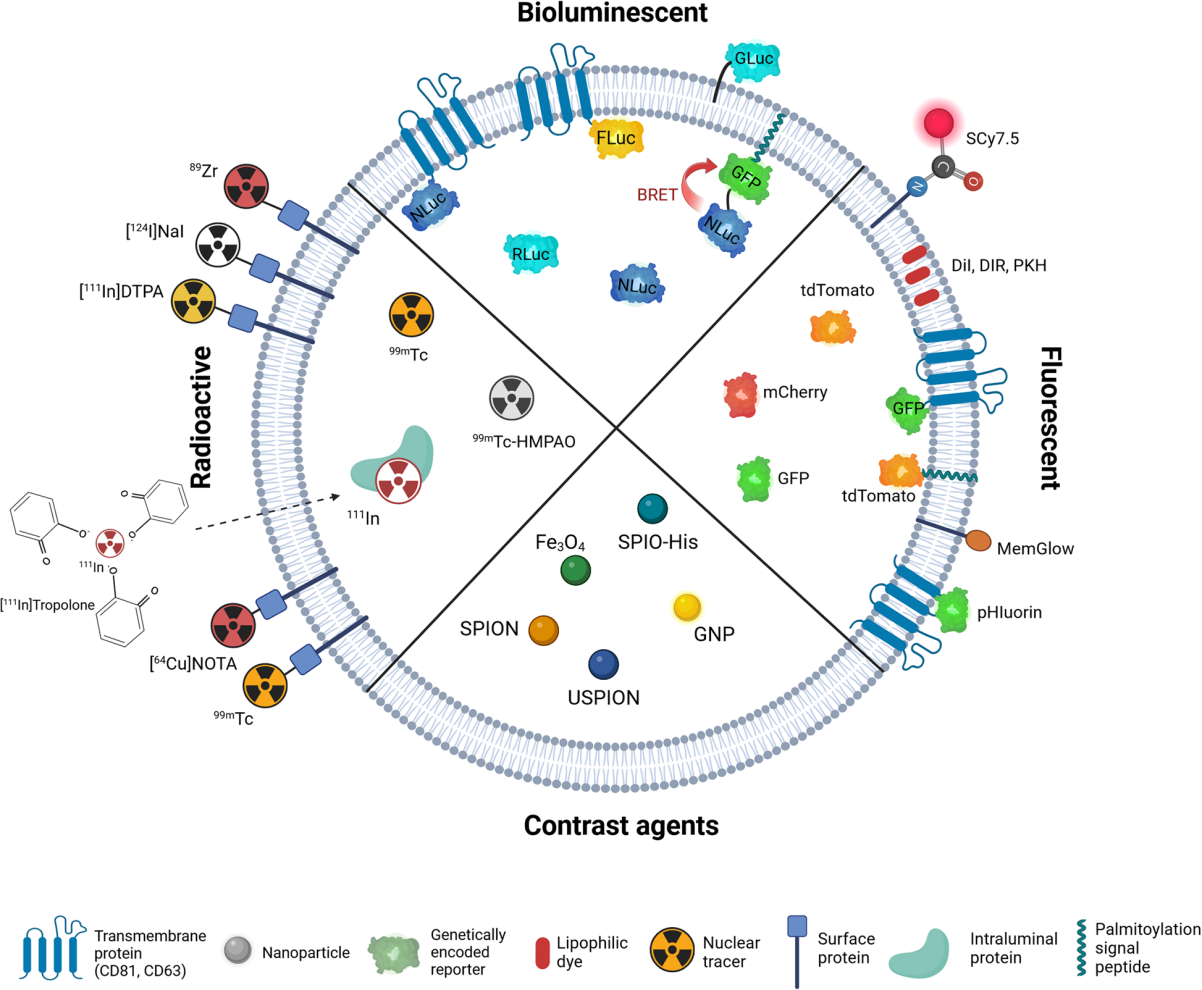
Strategies for labelling of exogenous extracellular vesicles

In this review, we discuss recent studies that explore labelling strategies aimed at both exogenously and endogenously produced EVs with an emphasis on methodology. In detail, we provide an overview of optical (fluorescence, bioluminescence), nuclear, and magnetic resonance imaging (MRI) tracers, and highlight their application for in vitro and in vivo EV tracking, for the study of the EV uptake mechanisms, or the tissue distribution after the administration of exogenously produced EVs into an animal model. The graphical overview of the labelling strategies for exogenously produced EVs is depicted in Fig. 1. Additionally, we review methods for monitoring direct functional uptake of endogenously released EVs and their tracking in vitro or in live animals. Finally, we evaluate the current advantages and limitations of each technique to discuss the future development of applications.

### Fluorescent imaging

Fluorescence is a widespread molecular and cellular imaging strategy for labelling and monitoring various biological structures in biomedical and analytical research. For fluorescence imaging, specimens are labelled with fluorophores, which emit fluorescence signals upon external excitation light. In the context of EV tracking, this method employs an array of various tools, including the expression of genetically encoded fluorescent protein reporters, lipophilic dyes incorporated into the lipid bilayer of the EVs, or the binding of fluorescent molecules that specifically attach to EV structures [12].

In relation to genetically encoded labels, gene constructs coding for fluorescent proteins are introduced into parent cells, which produce EVs that carry the desired fluorescent marker bound to their membranes. Lai et al. [13] engineered optical reporters to investigate the transfer of EVs and their RNA cargo between cells, both in cell culture and in an animal model. To label EV membranes, they used two constructs encoding either GFP or tdTomato protein attached to a palmitoylation signal. This strategy allowed them to label a broad population of EVs rather than just an EV subset with specific membrane markers, such as CD81. Utilizing two fluorescent reporters, they were able to confirm reciprocal EV exchange between two different cell lines and the EV uptake by recipient cells. Furthermore, they developed and tested a fluorescent reporter system that visualized co-localization signals between the EV membrane and its RNA transcript for efficient EV-RNA cargo monitoring. Importantly, they were able to track tumour-derived EVs from a mouse xenograft by intravital microscopy.

Already mentioned transmembrane proteins associated with EVs, including CD81, CD9, and CD63, have been extensively used for EV labelling. In the study by Lázaro-Ibánez et al. [14], they compared the efficiency of lipophilic DiR and mCherry-CD63 labelling strategy for in vitro and in vivo imaging. Tracking EVs tagged with mCherry by non-invasive imaging in tumour-bearing mice resulted in the detection of fluorescent signals in the abdominal and thoracic regions; however, the same areas were illuminated in control mice, which was confirmed by ex vivo analysis. These findings revealed that the high tissue autofluorescence within the excitation wavelength range of mCherry contributed significantly to the background signal, effectively reducing the signal-to-noise ratio and complicating the use of mCherry for precise EV monitoring. On the other hand, DiR labelling yielded a clear signal in the upper abdominal region corresponding to the liver and the spleen, while other organs or tumours had no detectable levels of fluorescence. Contrary to mCherry labelling, DiR provided an increased sensitivity, higher signal to noise ratio, and lower tissue autofluorescence, allowing for in vivo EV observation.

Apart from DiR, other commercial dyes such as DiI [15], PKH [16], and DiD [17] have been developed for direct lipid membrane labelling. However, it is important to note that their use is often limited only to in vitro applications. For instance, DiI and PKH dyes emit fluorescence at wavelengths between 500–570 nm [18, 19], which is not suitable for in vivo imaging. This is because shorter wavelengths are more prone to scattering and absorption by biological tissues [20], and also induce higher tissue autofluorescence [21], leading to reduced imaging clarity and contrast. On the other hand, dyes that emit in the near-infrared (NIR) spectrum, specifically in the 650–900 nm range, are better suited for in vivo imaging [22]. NIR light penetrates deeper into tissues with minimal scattering and absorption. Moreover, biological tissues exhibit significantly less autofluorescence in the NIR window, enhancing the signal-to-noise ratio, which is important for clear and accurate imaging [23]. The use of NIR dyes for in vivo imaging offers significant advantages for tracking and analysing EVs in complex biological environments. However, incorporating NIR molecules into EVs presents its own challenges. NIR dyes are more difficult to express in cells, and it is important to ensure that the labelling process does not alter the EVs’ natural characteristics or interfere with their biological functions.

In addition to emission wavelengths, the specificity of fluorescent dyes and their interaction dynamics are important for the reliability of EV labelling techniques. EV staining with lipophilic tracer is simply performed by the addition of the dye solution to the EV suspension with subsequent incubation and washing steps. This method, though practical for certain applications, does not discriminate between EV membrane and other lipophilic structures, potentially leading to non-specific labelling. Additionally, free dye molecules can form aggregates with sizes similar to EVs [24]. For example, Pužar et al. [25], demonstrated that in samples stained with the lipophilic dye PKH26, only 11% of fluorescent particles obtained by ultracentrifugation were labelled EVs, with the majority representing spontaneously formed PKH26 nanoparticles. Other experiments [26] showed co-isolation of large amounts of APOB^+^ lipoproteins with EVs and lipophilic dye transfer and its binding to non-EV components, with no correlation between the small EV content and dye uptake. Therefore, experiments including lipophilic dye staining should be carefully interpreted.

The development of novel dyes represents a significant advancement in overcoming the limitations of traditional lipid dyes. MemBright family of cyanine-based fluorescent probes avoid the formation of aggregates and remain non-fluorescent unless incorporated into membranes [27]. This characteristic allows more accurate and interference-free analysis in membrane-related studies, which is also applicable for EV monitoring. Loconte and colleagues [28] compared several labelling strategies including the use of MemBright to evaluate the best approach to monitor EVs uptake by recipient cells. The study proposed that passive and temperature-independent diffusion of the lipid-bound MemGlow-488 dye from the EV to the cell membrane could occur upon short or transient contact between the EV and the cell surface. This was in contrast to amine-reactive, cell-permeable dye CFSE and GFP reporter mp-sfGFP, where the signals were more likely to represent actual EV uptake and content delivery within the recipient cells. This suggests that some fluorescence signals might originate from dye transfer rather than actual uptake or content delivery of EVs. The authors also noted that the distribution of fluorescence from labelled EVs varied among different immune cell types within peripheral blood mononuclear cells. While MemGlow-488 was incorporated across all cell types, CFSE was observed in a minor subset of cells, and mp-sfGFP was mainly restricted to CD14^+^ cells. This indicates that the method of EV labelling significantly influences the detection and analysis of EV interactions with recipient cells.

Another approach how to prevent the possible detachment and release of lipophilic dye from the EV membrane was explored by González and colleagues [29], who utilised a chemical methodology that formed a covalent bond between the ester groups of a commercial lipophilic fluorophore and free amine groups of transmembrane proteins present in the EV membrane. In vitro experiments showed a dose-dependent cellular uptake of SCy7.5 labelled EVs after one hour of incubation with primary hepatocytes, with the strongest fluorescent signal detected after 24 h, when a high dose of dye was used. Additionally, higher solubility and an excitation wavelength near the infra-red region of SCy7.5 allowed its use for deep EV imaging in mice. Subsequent ex vivo analysis determined the highest fluorescent intensity in the liver tissue and weaker signals in the kidneys and the spleen.

A different approach to EV labelling was reported by Boysen et al. [30], who used helminths as an in vivo model to show the uptake of the fluorescent lipid analogue DOPE-Rho and its incorporation into EVs. As these worms are incapable of synthesizing fatty acids de novo, the pre-labelling of EVs was facilitated by the whole organism uptake of the fluorophore. The labelling method was further evaluated by adding fluorescently labelled EVs to activated Human Macrophage-Like THP-1 cells with subsequent overnight incubation. Fluorescent microscopy imaging confirmed a dose-dependent increase in the signal positive cells when DOPE-Rho labelled EVs were used. However, a higher dose of the lipid analogue was associated with reduced lipid uptake and a decrease in the number of EVs in the medium, suggesting potential toxicity.

Fluorescent proteins are useful tools for tracking exogenous EVs and monitoring intercellular communication in vitro or in animal models. However, their use poses some challenges, such as limited tissue penetration of emitted light, which is characteristic of fluorescent proteins with emission in the blue or green part of the spectrum. Moreover, non-invasive tracking is problematic due to the low resolution of the technique. Consequently, fluorescence imaging is often performed after an animal has been sacrificed or surgically exposed, limiting its use for real-time monitoring [31]. Additionally, producing fusion protein in excess can alter EV biogenesis, and its expression on the EV surface can create steric hindrance that may inhibit the EVs from binding to the target cell [32]. Despite these limitations, fluorescence can provide useful information about the in vitro and in vivo transportation of EVs and thus shed light on the biology of EVs.

### Bioluminescent imaging

Bioluminescence imaging (BLI) is based on the activity of luciferases, a family of oxidative enzymes that catalyse the conversion of specific substrates called luciferins to oxyluciferins, during which part of the chemical energy is converted into a photon of light. When compared to fluorescent imaging, luciferase enzymes produce a signal with low levels of background because they do not require an excitation source [33]. Bioluminescent reporters are derived from light producing organisms, including the marine *Gaussia princeps* (GLuc) or *Renilla reniformis* (RLuc), which have been used both for in vitro analyses to image EV release, their uptake by target cells, and for in vivo model systems that visualise tissue distribution.

**Fig. 2:**
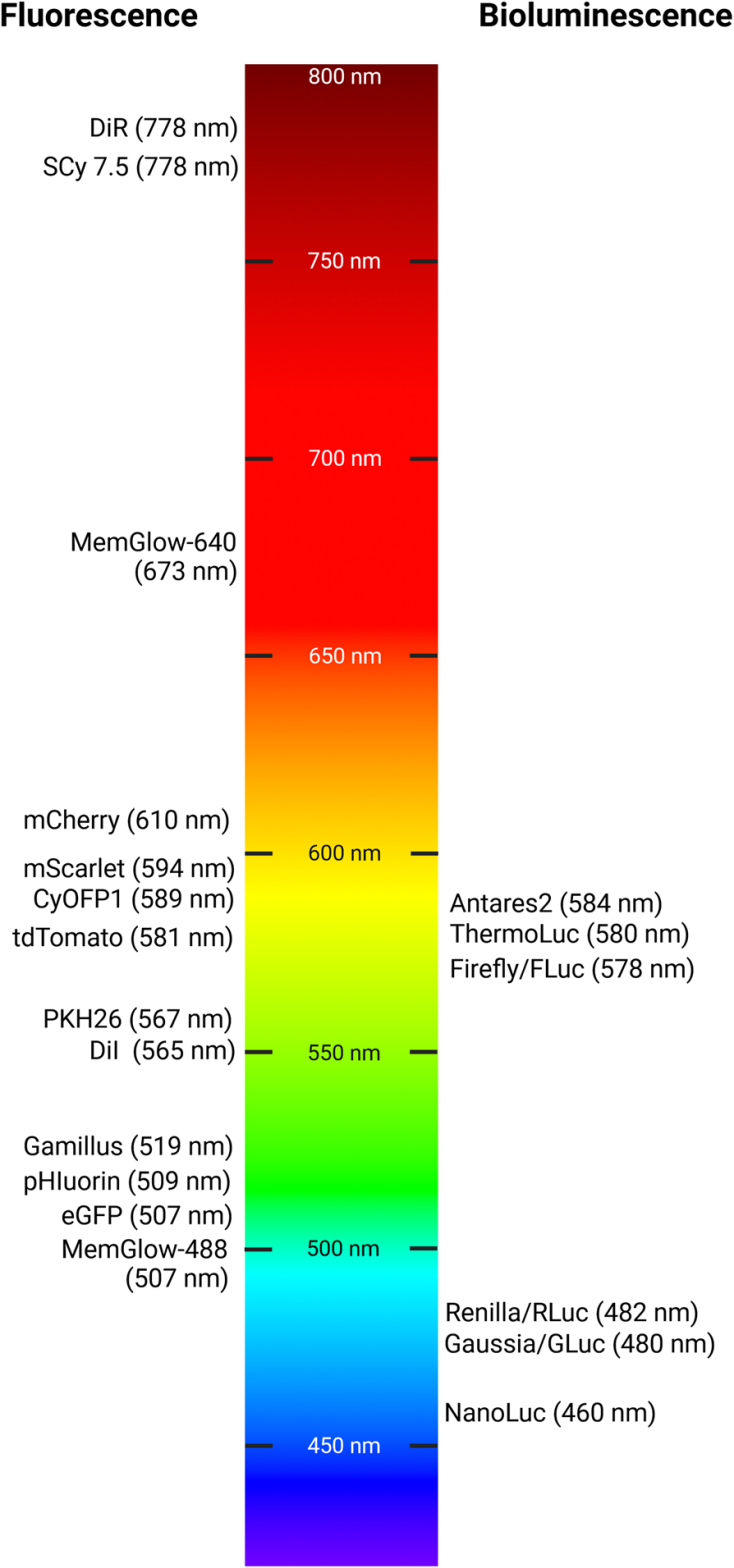
An overview of frequently used fluorescent and bioluminescent labels comparing their maximum emission wavelengths

Apart from their origin, luciferase enzymes differ in several chemical and photoluminescence properties that determine their use in preclinical studies and research. Emission wavelength is one of the crucial parameters to consider for non-invasive in vivo imaging because it limits how deep the emitted light can penetrate through mammalian tissues, with emission in the red part of the visible light spectrum providing deeper reach [34]. Figure 2 shows an overview of frequently used fluorescent and bioluminescent labels comparing their maximum emission wavelengths.

In addition to photoluminescent characteristics of luciferase enzymes, it is also essential to address the practical challenges of using these systems for EV tracking. A critical aspect is the substrate’s ability to effectively reach the accumulation site of EVs. Factors such as low substrate solubility [34], non-homogeneous substrate distribution or poor cell permeability [35–38] can significantly impact the effectiveness of EV monitoring in vivo.

Understanding the behaviour of endogenous and exogenous EVs in vivo is a prerequisite for the use of EVs as efficient drug delivery systems. For instance, EV clearance from the circulatory system is an aspect that has been explored in a study [39] that shows a quick capture of administered EVs by hepatic and splenic macrophages. In the experimental setup, EVs from murine melanoma cells were labelled with GLuc-lactadherin and intravenously injected into macrophage-depleted mice to demonstrate higher EV concentrations both in the serum and in organs such as the liver, spleen, and lung, when compared to untreated mice four hours after administration.

Gupta and his colleagues [40] compared the sensitivity, stability, and luminosity of five different luciferase enzymes fused either to the N- or C-terminus of tetraspanin CD63 that belongs to the transmembrane protein family. The NanoLuc (NLuc) enzyme alone or its fusion to either of the terminal tails showed the highest luminescence and stability, followed by the ThermoLuc CD63 C-terminal fusion, while Super RLuc8, Firefly and CRBLuc CD63 tethering produced lower quantum yield. For in vitro applications, the authors used CD63-ThermoLuc labelled EVs to show their quantitative uptake in recipient cells, which occurred in a dose-dependent manner. Furthermore, the in vivo application allowed them to observe the pharmacokinetic profile and biodistribution of CD63-NLuc engineered EVs in mice. Their study demonstrated rapid plasma clearance of exogenously administered EVs mainly in the liver and spleen five minutes post injection, with a gradual decrease over time. Interestingly, the authors showed that the difference in EV biodistribution depended on the route of administration, where intravenous and peritoneal injection demonstrated a similar pharmacokinetic profile in circulation while subcutaneous application indicated a lower luciferase activity in mouse plasma. Additionally, different arterial or venous applications resulted in similar systemic distribution, contrary to the peroral or intracerebral injection that limited the EV dispersion primarily to the stomach and brain, respectively. For non-invasive tracking, they used ThermoLuc labelled EVs that distributed mainly to the lungs, liver, and spleen. Importantly, a significant difference in body-wide distribution pattern was also observed for different EV subpopulations when the luciferase was fused to either CD63 or CD9, manifesting enhanced accumulation of EVs in the gastrointestinal tract or lungs, respectively.

In the study conducted by Lázaro-Ibánez et al. [14], the authors compared five different optical and nuclear tracers to elucidate the effect of labelling on EV properties, its efficiency and EV biodistribution pattern in vivo. When comparing the two luciferase systems, NLuc provided a 10^5^-fold brighter signal per particle than the firefly luciferase (FLuc), and it was sensitive enough to detect 10^5^ − 10^6^ of bulk EVs in vitro. The labelling efficiency of NLuc was also higher than that of Fluc, and it manifested as a strong bioluminescent signal after cellular uptake. Furthermore, in vivo imaging performed one and four hours after the administration of exogenous EVs showed pale signals in the area of the spleen. Although ex vivo EV detection provided a stronger quantum yield than in vivo bioluminescence, the tissue distribution of injected NLuc-EVs was partially different from the biodistribution of the other tested labels. While the strongest NLuc signals were recorded in the lungs and spleen with a weaker signal emitting from the liver, fluorescent DiR or radioactive [^111^In]-DTPA tracer showed the highest accumulation in the liver and the spleen. The authors hypothesized that the change in the distribution pattern could be caused by a change in the surface protein composition following the expression of an exogenous fusion protein.

### Dual-reporter systems

Although ex vivo measurement of NLuc activity is effective for determining EV biodistribution after their administration, it is not a suitable method for long-term EV monitoring. Therefore, Hikita et al. [41] utilised the bioluminescence resonance energy transfer (BRET) reporter Antares2 that is based on a mutated NLuc luciferase fused with the fluorescent protein CyOFP1 as an acceptor chromophore to visualize the biodistribution of cancer-derived EVs. The bioluminescent reporter was attached to CD63 to ensure efficient EV loading. For EV tracking, cancer cells were engineered to express the CD63-Antares2 construct, and these genetically modified cells were introduced subcutaneously into mice. The presence of Antares2 allowed for the detection of bioluminescent signals in blood samples, taken at five-day intervals. Subsequent analysis of blood samples revealed that BRET signal intensity increased with the growth of the tumour, indicating the accumulation of EVs in the bloodstream. Due to the low spatial resolution of whole-body imaging, ex vivo bioluminescent imaging was performed, resulting in signal detection in various organs, namely, the lungs, stomach, intestine, and genital glands.

Wu and his colleagues [42] created another modification of the BRET system called PalmGRET by fusing a palmitoylation signal peptide sequence from the GAP43 protein to the N-terminus of a GFP-NLuc BRET reporter, which enabled multi-resolution visualization of EVs by live cell microscopy and in vivo imaging system. After intravenous application of EVs in mice, their highest concentrations were observed in the region of the lungs and liver.

Analogous variation of the BRET system was reported by Perez et al. [43], who utilised red-shifted PalmReNL which is based on tdTomato-NLuc fusion protein. Confirmed by flow cytometry, the labelling efficiency of PalmReNL was 1.2% and 6.3% for small EVs (sEVs) and medium/large EVs, respectively. Surprisingly, reporter sEVs did not emit fluorescence after uptake into recipient cells, contrary to the mScarlet-based reporter fused to CD63 that sustained the fluorescent signal after cellular uptake of sEVs. The authors speculated that the observed difference in fluorescence retention could be caused by diverse cell processing of the two reporters, or possibly, by preferential labelling of distinct sEV populations. Additionally, in vitro testing of the reporter’s susceptibility to an acidic environment resulted in a significant reduction of the fluorescent signal of PalmReNL-sEVs after cellular uptake, compared to the acid-tolerant Palm-fused Gamillus, whose signal remained easily detectable after 24 h. Furthermore, a gradual decrease was observed for bioluminescent signals when small and medium/large EVs carrying PalmReNL were exposed to pH below six that is common for late endosomal and lysosomal compartments. Testing in vivo and ex vivo biodistribution after retro-orbital administration in mice showed higher bioluminescent signal for small PalmReNL EVs in comparison to medium/large EVs. Moreover, the use of a novel, more soluble substrate called fluorofurimazine yielded a stronger signal than furimazine both in vitro and in vivo.

Due to low spatiotemporal resolution of the bioluminescent reporters and poor substrate distribution or solubility [44], non-invasive EV tracking is challenging, and the accurate anatomical information is often limited to ex vivo analysis. Nonetheless, modified reporters with enhanced signal intensity, novel substrates, increased emission wavelength, or their combination with fluorescent BRET acceptors have overcome most of the limitations by offering better imaging sensitivity and higher tissue penetration.

### Radiolabelling

Nuclear imaging of EVs incorporates a range of techniques that use radioactive labels detected with Single-Photon Emission Computed Tomography (SPECT) or Positron Emission Tomography (PET). The main advantages of these methods include the wide availability of radionuclides and the preservation of EVs’ morphology after labelling [45]. SPECT imaging utilizes gamma-emitting radionuclides that emit single photons with half-lives ranging from hours to days. Detection of emitted photons is secured by a rotating camera that generates a 3D image. Some radionuclides commonly used in this technique include Iodine-123, Technetium-99m, and Indium-111 [46, 47]. On the other hand, PET imaging is based on positron-emitting radionuclides. These radioisotopes emit positrons that annihilate nearby electrons, releasing a pair of gamma rays of equivalent energy in opposing directions. Gamma rays are then detected by a ring of detectors, mapping the incoming radiation at a 180° angle, a process known as coincidence detection. Zirconium-89, Copper-64, Gallium-68, and Fluorine-18 are among the radionuclides typically employed for PET imaging, which offers approximately two to three times higher resolution than SPECT [45, 47]. Both SPECT and PET are imaging methods suitable for visualizing the movement and quantifiable changes of EVs in vivo, and they are often used in conjunction with CT or MRI scans for precise anatomical localization [48, 49]. Various radionuclide labelling strategies are employed for EV tracking, however; depending on the localization of the radionuclide within the EV, these strategies can be primarily categorized as intraluminal or surface labelling. Surface labelling involves the attachment of radionuclides to the EV membrane either by covalent bonding of the tracer to a membrane receptor or via the use of chelating agents [50].

Royo et al. [51] investigated the impact of manipulating EV glycosylation, a key protein modification affecting membrane-to-membrane interactions, on EV distribution pattern. In their study, EVs were first treated with neuraminidase to cleave sialic acid residues from glycoproteins, and then were directly labelled with [^124^I]Na. Modified membrane glycoproteins showed an increased accumulation of EVs in the lungs and axillary lymph nodes of mice when compared to EVs that were not enzymatically treated, suggesting that manipulation of the glycosylation pattern can affect EV biodistribution. Additionally, PET imaging allowed for quantitative monitoring of [^124^I]Na labelled EVs for up to 72 h.

Another labelling strategy utilizes a chelating agent to create a link between radionuclide and the membrane, which was reported in a study by Lazaro-Ibáñez et al. [14]. The authors used covalent linking of DTPA‑anhydride to the EV surface, which functioned as a bifunctional chelator for labelling EVs with ^111^In^3+^. This method demonstrated high EV radiolabelling efficiency and increased stability in 50% serum. The combination of SPECT with anatomical data obtained from CT provided high sensitivity and deep tissue penetration, suitable for tracking ^111^In^3+^DTPA labelled EVs in mice. As opposed to optical approaches, the radioactive signal in excised organs was measured without the need for further tissue homogenization.

Direct labelling of EVs is often viewed as a standard approach. However, the use of certain radionuclides that require harsh conditions, such as high temperatures or strong bases, could potentially damage EVs. In contrast, indirect labelling utilizes prosthetic groups that can withstand the severe conditions necessary for their association with the membrane [50]. One of the prosthetic groups is 1,4,7-triazancyclononane-1,4,7-triacetic acid (NOTA), which works well as a bifunctional chelator for radiolabelling. Shi et al. [52] used the label in combination with radioisotope ^64^Cu and compared the in vivo properties of NOTA-^64^Cu EVs versus equally labelled EVs with added polyethylene glycol (PEG) on their surface. Their radiostability experiments showed that a higher percentage of the NOTA-PEG-^64^Cu label was retained in mouse serum at 24 h compared to NOTA‑^64^Cu EVs without PEGylation. After intravenous application in mice, PEGylated EVs were less prone to clearance by the liver and exhibited enhanced tumour uptake than ^64^Cu-NOTA-EVs.

Another indirect surface labelling was explored by Molavipordanjani S. et al. [53], who utilised *fac*-[^99m^Tc(CO)_3_(H_2_O)_3_]^+^ reactive group to radioactively label EVs that express DARPin (designed ankyrin repeat proteins) G3 ligands on their surface with an affinity to HER2 tyrosine kinase receptors. In vitro experiments demonstrated an increased binding of ^99m^Tc-EVs to SKOV-3 cancer cells that had higher HER2 expression than in other tested cell lines. Additionally, active HER2 targeting was evaluated in a SKOV-3 xenograft mouse model, demonstrating an accumulation of EVs in the tumour tissue and indicating the potential use of radioactive nanoparticles in tumour imaging or treatment.

Intraluminal labelling refers to the incorporation of the radioactive molecules within the lumen of EVs. This can be achieved through various methods such as passive diffusion, active loading via membrane transporters, electroporation, or sonication. This approach is typically used when surface labelling would alter the EV membrane [45, 50].

While imaging techniques using radioactive ions have significant capabilities, the efficiency and stability of radiolabelling can be low in some cases. For instance, Faruqu et al. [48] aimed to label EVs with ^111^In^3+^ using tropolone, a hydrophobic chelating molecule that can diffuse across the EV membrane in complex with a radionuclide. However, the labelling efficiency was lower than expected, likely due to the lack of biomolecules in the EV lumen available for ^111^In^3+^ exchange, which allowed the [^111^In]tropolene complex to diffuse back to the extravesicular space.

The tracking of EVs in vivo has emerged as a valuable tool for studying their migration and localization. The articles referenced in this review report a comparable distribution pattern, with the strongest signals detected in organs primarily responsible for the clearance of substances from the body, such as the liver, kidneys, and spleen [49, 52, 53]. Importantly, the cited studies offer strategies for molecular imaging by PET and SPECT, which will be important for future applications of EVs in diagnostics and therapeutic delivery systems [53]. However, several challenges to consider include the possibility of changes in EV characteristics after radionuclide application, the high cost of labelling tracers, regulatory policies for using radioactive material, and the need for trained personnel to handle these substances [31].

In recent years, the application of radionuclide labelling techniques for in vivo tracking of EVs has gained increasing attention. Imaging techniques PET and SPECT have significantly advanced our understanding of EV migration, accumulation, and biodistribution. However, the integration of these techniques with structural imaging modalities such as CT and MRI can further enhance the functional information with high-resolution anatomical details and offer more comprehensive understanding of EV dynamics within the body.

### Magnetic resonance

Magnetic Resonance (MR) imaging is a technique based on the response of hydrogen nuclei to radio-frequency pulses in the imaged tissue. This response varies depending on the MR properties of the tissue, such as relaxation times, spin density, chemical shift, and molecular motion [54, 55]. By using different MR pulse sequences, MRI can offer varied image contrasts, reflecting the tissue properties. Apart from the established use in detailed tissue imaging, these contrast mechanisms in MRI can also be applied to tracking and monitoring EVs in vivo, allowing their enhanced visibility in biological systems.

The use of MRI in vivo was presented in the study by Hu et al. [56], who inserted superparamagnetic iron oxide nanoparticles (SPION5) into EVs using electroporation, enabling their tracking in vitro and in an animal model. The authors observed that the injection of SPION5 EVs, as well as free SPION5 into the mouse footpad, caused the enlargement of the popliteal lymph node, with a significant deposition of SPION5 EVs after 48 h compared to the SPION5 application alone. Interestingly, EVs derived from melanoma cells were preferentially taken up by the subcapsular region of the mouse lymph node, suggesting the potential use of therapeutic exosomal inhibitors in the future, that could prevent the formation of a premetastatic niche mediated by tumour-released EVs.

A different labelling technique that could also assist in EV-based drug delivery was introduced by Han et al. [57], who loaded superparamagnetic iron oxide particles conjugated with a histidine polypeptide (SPIO-His) into purified EVs. When compared to an alternative method for SPIO labelling described by Busato et al. [58], the electroporation of SPIO-His particles improved the efficiency, achieving a rate of 96% versus 19%, when SPIOs were incubated with parental cells to produce SPIO-labelled EVs. Moreover, the use of magnetic particles simplified the enrichment of EVs through the application of an external magnetic force and additional purification using a Ni–NTA column enabled the separation of encapsulated particles from free SPIO-His labels. In a rodent model of acute kidney injury, the systemic administration of magnetic EVs generated quantifiable MRI signals, which revealed a significantly increased uptake of iPSC-derived vesicles in the injured kidneys compared to controls. The enhanced survival rate of mice and the protective effect on tissues indicate the possible future applications of magnetic EVs in the optimization of systemically administered therapies.

As mentioned previously, Busato and colleagues [58] developed a different method for magnetic particle packing into EVs which involved cultivating adipose stem cells (ASCs) with an iron-based USPIO label. The primary objective was to adopt an alternative, non-invasive approach for EV labelling without disrupting the membrane characteristics. The authors identified an optimal concentration of USPIO that enabled the labelling of ASCs with a complete efficiency over a 72-h incubation period, without any reduction in their viability. Confirmed by TEM imaging, USPIO nanoparticles were also present in EVs isolated from conditioned ASC medium. Following an intramuscular application in mice, the team found that labelled EVs were readily detectable by MR imaging, which was also validated by histological examination.

Shaikh et al. [59] adopted an alternative strategy for the synthesis of nanoclusters with multimodal properties. They introduced solutions of IrCl_3_ and FeCl_2_ to either cell culture media or mice with tumour xenografts. This led to the in situ formation of luminescent IrO_2_ and magnetic Fe_3_O_4_ in the tumour tissue, acting as imaging probes with an increased image sensitivity, biocompatibility and tumour specificity. The process was enabled by reducing agents naturally present in the tumour microenvironment, which exhibits different redox homeostasis than normal tissue. Interestingly, EVs extracted from the serum of mice bearing tumour xenografts—but absent in EVs from control mice—contained these newly formed nanoclusters, suggesting their potential use as cancer biomarkers.

The implementation of MRI is becoming increasingly frequent due to its capacity to deliver detailed anatomical images and accurate spatiotemporal data about the label [60]. In addition, it provides high contrast for soft tissue imaging, and the MRI reagents being non-radioactive have an extended shelf life. However, MRI presents some disadvantages, which include the high cost of the reagents and the MRI instrumentation itself. Furthermore, MRI cannot be performed on individuals with metallic or magnetically sensitive implants [61]. While MRI is often used in combination with other techniques such as PET and SPECT, ongoing research and technological advances are enabling its use to offer comprehensive imaging of EVs without the need for complementary techniques [58].

### Computed tomography/Xray imaging

Computed tomography (CT) is a technique that uses an X-ray emitter and a row of scan receptors placed on the opposite side to make measurements of X-ray attenuation caused by tissue density. These measurements make 2D images of the subject from different angles, and once all the slices are combined, a tomographic image will present pictures of the whole body or a particular organ [61].

As mentioned in the chapter about radiolabelling, the primary role of CT in tracking EVs is to supplement PET or SPECT scan by providing comprehensive anatomical details, as in Varga et al. [49], where EVs derived from human erythrocytes were labelled with ^99m^Tc-tricarbonyl complex for in vivo monitoring in mice. After EV labelling, the combination of CT and SPECT imaging data allowed for the precise localisation of the radiolabelled EVs within the body. An example of EV tracking by PET/CT can be found in Choi et al. [62], who intravenously administered ^89^Zr-labelled EVs in mice and rats to determine their quantitative biodistribution, pharmacokinetics, and excretion rate in different animal models.

Nonetheless, CT can also be used independently for EV research, such as in Cohen et al. [63], where EVs derived either from mesenchymal stem cells (MSC-exo) or from the A431 squamous cell carcinoma line (A431-exo), were loaded with gold nanoparticles for non-invasive CT imaging. The team reported an infiltration of gold-labelled EVs to the tumour tissue and cell cytoplasm, while the free gold particles were retained at the tumour periphery. The results revealed quantitative differences in the vesicle uptake manifested by higher concentration of MSC-exos in tumour after 48 h when compared to that of A431-exos. The authors concluded that the source of EVs significantly influenced their capacity to target and infiltrate tumours, and it could have potential implications in EV-mediated cancer treatments.

In the study conducted by Lara et al. [64], the team developed a method to indirectly label melanoma EVs with gold nanoparticles (AuNPs), while preserving their inherent characteristics. They incubated B16F10 melanoma cells with folic acid-conjugated AuNPs, which enhanced the labelling efficiency and facilitated the integration of AuNPs into EVs. The results showed that murine melanoma cells exhibited a preferential uptake of their own EVs, and also suggested the potential of using EVs as a targeted drug delivery system for the treatment of metastasis. Importantly, the study demonstrated that a single administration of either AuNPs, EVs, or EV-AuNPs did not significantly stimulate tumour growth, highlighting the potential safety of this approach. Additionally, pairing CT imaging with fluorescent assays and optical microscopy allowed visualisation and quantification of gold uptake by different tissues.

CT imaging has significantly evolved from its initial role as a supporting tool for EV analysis performed by other methods. Recent studies have demonstrated its effectiveness for in vivo tracking of EVs and whole-body imaging. However, the technique poses some challenges associated mainly with ionizing radiation. Although the dose of ionizing radiation has been reduced over the years, CT scans are not recommended during pregnancy and frequent imaging should be avoided for the same patient. Despite these limitations, CT scanning offers several important benefits. These include direct detection of tracers, deep tissue penetration, and the capacity to image the heart and gastrointestinal tract without interference from gas pockets or artifacts [65]. Additionally, the imaging agents used in CT scans are stable with a long shelf-life.

Effective labelling and tracking of exogenous EVs present distinct challenges across various techniques. Fluorescent and bioluminescent imaging offer detailed insights but face limitations in vivo due to autofluorescence and light scattering or enzyme substrate variability. Radiolabelling, while sensitive, is limited by potential EV alterations and regulatory obstacles. MRI and CT imaging provide detailed anatomical data but come with high costs and technical limitations. Ultimately, surface modification methods like NHS chemistry and cargo loading approaches such as electroporation, though widely used, risk altering EV surface protein functionality and can lead to EV malformation. These issues impact the biological roles and therapeutic potential of EVs. To conclude, the key challenge in EV labelling and tracking is maintaining a balance between effective labelling and preserving the natural characteristics of EVs. Advancements in this area are needed for the accurate study of EV behaviour and their potential therapeutic applications.

### Monitoring the uptake of endogenously released extracellular vesicles

The majority of published studies utilise exogenously administered EVs in animal models to evaluate their tissue distribution, typically using in vivo/ex vivo EV tracking reporters. However, only a small fraction of recent literature reports the transfer of endogenously produced EVs to their recipient cells or tissues, thus reflecting more realistic observations of concentration, location, and nature of continuous EV release. In this context, the use of Cre-loxP recombination and CRISPR-Cas systems has emerged as a powerful approach, not only for tracing the uptake of endogenously formed EVs in vitro and in vivo, but more importantly, for monitoring the successful delivery of specific EV-encapsulated cargo into the cytoplasm of target cells. This distinction is important, considering the challenges associated with achieving high loading efficiency of therapeutic compounds in EVs and the fact that EV uptake does not necessarily equate to effective intracellular cargo delivery. Importantly, these genetic tools provide a more accurate representation of EV-mediated cargo transfer, rather than simply indicating EV tropism.

**Fig. 3:**
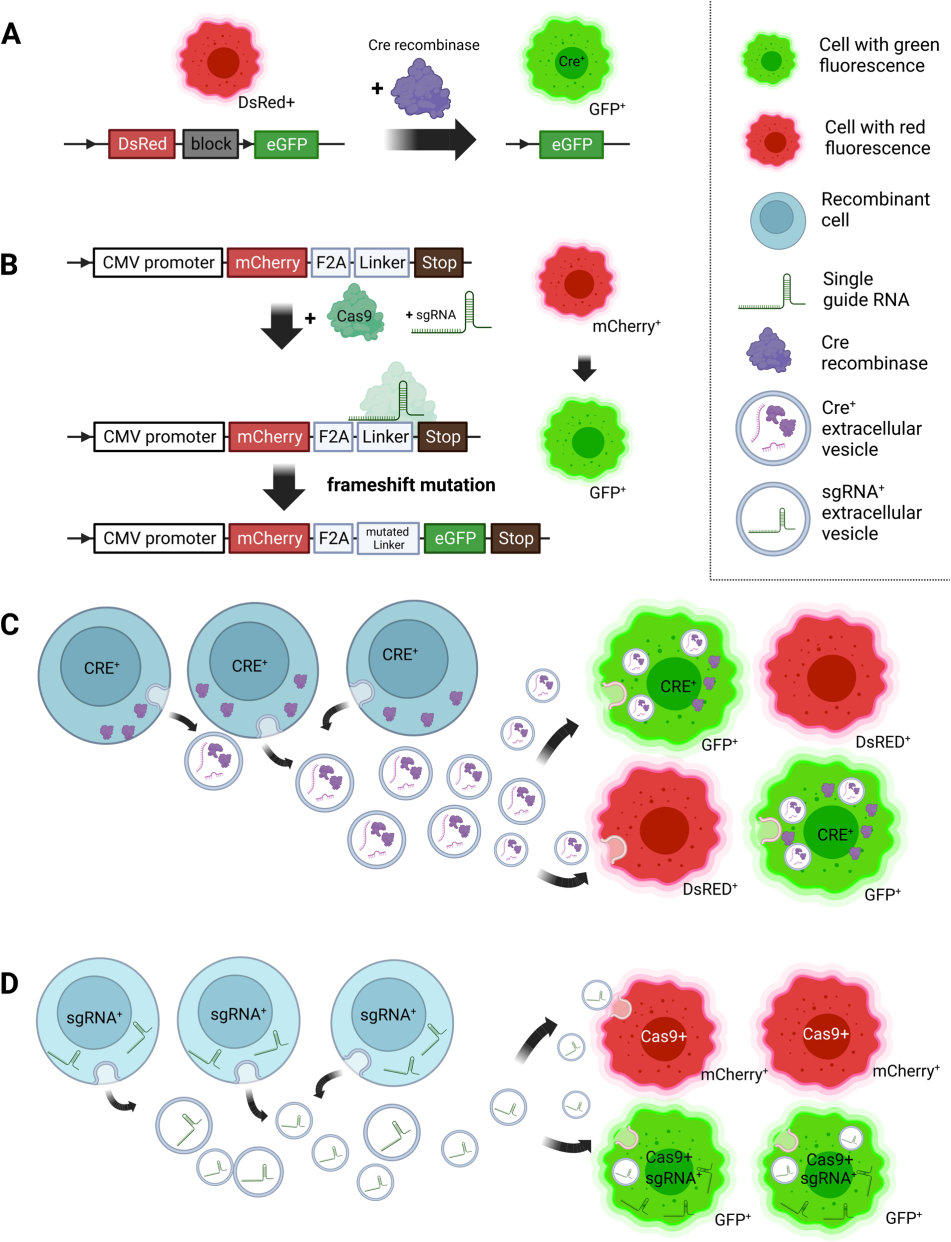
Strategies for the labelling of endogenously produced EVs for the study of their functional transfer. **A** Cre-LoxP method. **B** CRISPR-Cas9 method. **C** Principle of Cre-LoxP system: Cre-mediated recombination activates a fluorescence switch from DsRed to eGFP in reporter^+^ cells after receiving Cre^+^-EVs produced by Cre^+^ cells. **D** Mechanism of CRISPR-Cas9: Reporter^+^ cells expressing Cas9 switch from mCherry to eGFP fluorescence after functional uptake of EVs. EVs produced by genetically manipulated donor cells carry a specific targeting single guide RNA (sgRNA) that navigates Cas9 with nuclease activity to generate cleavage of the target sequence

The mechanism underlying Cre-LoxP recombination in the context of EV cargo delivery is based on cells expressing Cre recombinase (Cre^+^ cells) and cells carrying Cre reporter (reporter^+^ cells) that would mark EV-mediated Cre transfer by emitting fluorescence or bioluminescence (Fig. 3A and C). For in vitro experiments, the Cre-LoxP method can be used for analysis and quantification of EV content exchange between different cell types when the Cre^+^ cells and reporter^+^ cells are either cocultured or are physically separated by a transwell membrane that allows only EVs to cross. Moreover, adding purified Cre^+^ EV preparations to reporter^+^ cells offers a possibility to study a differential functional effect on the target cells. In mice, the study of EV uptake can be facilitated using transgenic Cre^+^ and reporter^+^ animals or by administering both Cre^+^ and reporter^+^ cells together in the same mouse [66].

To elucidate if the recipient cells change their behaviour after uptake of in vivo-released tumour EVs, Zomer and colleagues [67] employed Cre-LoxP system to activate a fluorescence switch from DsRed to eGFP in reporter^+^ cells after receiving EVs produced by Cre^+^ cells. The in vitro experiments confirmed functional EV-mediated Cre transfer and showed an increase in eGFP^+^ reporter^+^ cells with a higher ratio of Cre^+^/reporter^+^ cells using confocal microscopy. Interestingly, cell-to-cell contact-independent Cre transfer was also observed between malignant (MDA-MB-231) and less malignant cells (T47D), indicating a functional transfer of biomolecules, which could affect the cell physiology and behaviour. Analogous to their in vitro observations, orthotopically transplanted reporter^+^ cells forming metastatic tumours in the mammary glands of mice could take up Cre^+^-derived EVs that were injected into the tumour and report the Cre activity as eGFP^+^ cells. Similarly, a Cre mediated red-to-green colour switch was observed in tumours consisting of Cre^+^ and reporter^+^ cells. By administering Cre^+^ B16 melanoma cells to form tumour in mice that expressed the Cre-LoxP reporter tdTomato, the team could observe non-tumour cells expressing tdTomato in various tissues, which suggested transfer of EVs between tumour and healthy cells. However, the reverse mechanism – the uptake of EVs from healthy cells by melanoma cells – did not occur frequently. Further experiments, performed by multi-photon microscope, allowed for intravital imaging, which showed enhanced migration of T47D cells when more malignant cells were in close proximity, compared to when they were located in a distant tumour. Moreover, the analysis of lung metastatic lesions and primary tumours revealed a 52-fold and eightfold increase in the metastatic potential of T47D cells, demonstrated by eGFP positivity upon local or distant communication with MDA-MB-231 cells, respectively, indicating EV-mediated spread of metastatic behaviour.

A modification of Cre-LoxP system for in vitro and in vivo applications was demonstrated by Luo et al. [68], who utilised CD63 fused NLuc reporter, whose expression was under the control of a tissues-specific αMHC promoter to visualise and track endogenous EVs produced by transgenic mouse cardiomyocytes. Additionally, inserting LoxP-STOP-LoxP sequence for inducible expression of the CD63NLuc reporter allowed temporal EV labelling and monitoring in vivo. The tissue uptake of cardiomyocyte-derived EVs was analysed ex vivo, with significant luciferase activity recorded in the thymus, testis, lung, and kidney, and minimal signals in the other nine organs tested. In comparison, biodistribution of free NLuc administered to mice manifested primarily in the liver.

Similarly, CRISPR/Cas9 technology is used for gene editing in eukaryotic organisms employing single guide RNA (sgRNA) that navigates Cas9 with nuclease activity to generate cleavage of the target sequence, to which guide RNA is complementary [69]. In a recent study, de Jong et al. [70] designed a novel approach to study functional intercellular RNA exchange by the CRISPR-Cas system, which involved the EV-mediated transfer of sgRNAs expressed in EV donor cells. Upon the functional uptake of a specific targeting sgRNA to the EV-acceptor cells expressing Cas9 and the “stoplight” reporter, the fluorescent reporter is permanently activated indicating successful cargo transfer between the cells. The reporter system is based on the constitutive expression of mCherry that switches to permanent eGFP fluorescence after functional uptake. The switch is triggered by Cas9-mediated double-stranded breaks, followed by NHEJ-mediated repair, frameshifting, and evasion of the original stop codon (Fig. 3B and D). The stoplight system was tested in vitro; co-cultivation of reporter and sgRNA^+^ cells resulted in a dose-dependent eGFP activation, albeit with a low activation rate (up to 0.2%). By using transwell coculture assay, the authors also confirmed that cell-to-cell contact is not required for the functional sgRNA transfer. Additionally, administering the EV separated from the donor-cell conditioned medium to reporter cells resulted in significant eGFP activation. Finally, the stoplight reporter workflow was utilized for elucidating the role of various regulatory genes in functional RNA delivery by EVs.

The use of the CRISPR/Cas9 system for studying EV-mediated cargo delivery and processing was also reported by Ye and colleagues [71], who investigated the transfer of tumour-derived EVs from a donor cell line expressing both sgRNA pairs and Cas9 proteins. The STOP-fluorescent protein expression in recipient cells ensured the induction of GFP signal upon functional EV uptake. To increase the loading of Cas9 into EVs, they fused mCherry nanobody to Cas9 and mCherry to CD63, respectively. By co-culturing donor and recipient tumour cells or adding donor-derived EVs to recipient cells, the authors observed the EV-mediated cargo marked by green fluorescence. Interestingly, culturing tumour cells with a non-tumour cell model yielded similar effects, indicating that EVs released from tumour sgRNA:Cas9^+^ cells could be taken up by normal cells. Besides in vitro experiments, the CRISPR-Cas9 system was also adopted in an animal model that ubiquitously expressed the STOP-tdTomato sequence, targeted by Cas9 nuclease and delivered by EVs. The repeated administration of EVs resulted in red fluorescent signals in the liver, while endogenously produced EVs, derived from engrafted donor B16 melanoma cells, were additionally detected both in the liver and the brain. The authors speculated that EV integrins could be responsible for the organ tropism, such as liver targeting ITGβ5 that was abundantly present in B16 EVs.

CRISPR-Cas and Cre-LoxP systems are available tools for monitoring endogenously produced EVs, and in the case of tumour-released EVs they can elucidate the functions of EVs in tumorigenesis with insight into organotropism and tissue invasion of metastatic cancer. Possible complications could originate from the use of transient DNA-transfection agents as free plasmids are hard to remove from the EV preparation, thereby eliciting a false positive fluorescent signal [71]. Additional limitation includes the permanent activation of the reporter gene after the functional EV uptake, with cumulative effects that do not reflect to real-time EV tracking [72]. However, despite these drawbacks, the use of CRISPR-Cas and Cre-LoxP methods can assist in deciphering the roles of EVs in cell communication, in understanding EV signalling under physiological or pathological conditions, and in adopting improved EV-mediated therapeutic targeting.

While CRISPR-Cas and Cre-LoxP systems facilitate the monitoring of endogenously produced EVs, it is essential to differentiate between the successful delivery of their encapsulated cargo and the general uptake and biodistribution of EVs. Studies like the one conducted by Verweij et al*.* [73] focus on understanding EV tropism and biodistribution, to elucidate how EVs target specific tissues and distribute within the body. Specifically, the authors developed a novel in vivo model using zebrafish embryos expressing CD63-pHluorin. This fluorescent reporter targeted to cell (late-) endosomes allowed for the detailed investigation of the release, transfer, and uptake of endogenously secreted exosomes. The study identified a subpopulation of EVs with exosome characteristics, released from the yolk syncytial layer (YSL) into the blood flow, which could be monitored up to their target destination. These exosomes were taken up, and degraded by macrophages and endothelial cells in the caudal vein plexus (CVP), highlighting a functional inter-organ communication by exosomes. The study revealed that the uptake of YSL-derived exosomes by endothelial cells is mediated through scavenger receptors and dynamin-dependent endocytosis, and the manipulation with exosome biogenesis affected CVP growth, supporting a role of these exosomes in providing trophic support to the endothelial cells.

Scott et al. [74] also utilised zebrafish model, but their research was aimed at EVs in cardiovascular biology. The study focused on active production of EVs by various cell types, including endothelial cells and cardiomyocytes, in both larval and adult zebrafish. This was achieved using stable transgenic zebrafish lines expressing prenylated mCherry fluorophore driven by cell specific, cardiovascular relevant promoters. This novel system allowed for the fluorescent labelling and in vivo tracking of endogenously secreted EVs. High spatiotemporal resolution light-sheet live imaging and modified flow cytometry methods were employed for cell-type specific EV tracking and detailed analysis. The authors observed exchange of EVs between different cell types in the adult zebrafish heart. Furthermore, the study demonstrated that ischemic injury models can dynamically alter EV production, indicating a responsiveness to pathological conditions. Additionally, the study provided insights into the tropism of EVs, suggesting their targeted interaction and communication within the cardiovascular system.

The behaviour of tumour-derived extracellular vesicles (TEVs) in metastasis, particularly in the context of pre-metastatic niche (PMN) formation was studied by Blavier et al. [75]. The study explored the endogenous release of GFP-tagged TEVs from metastatic human melanoma and neuroblastoma cells and their specific capture by macrophages and stromal cells in various mouse organs. This early capture occurred before the homing of metastatic tumour cells, highlighting the significant role of TEVs in PMN formation. This organotropic uptake of TEVs significantly influenced inflammatory gene expression in these cells, contributing to the development of a pro-tumorigenic environment.

## Conclusions

In our review, we have discussed the benefits and limitations of methods used for EV labelling and for monitoring their fate in a Petri dish or in a live animal to aid the selection of a suitable labelling method.

Interestingly, the in vivo biodistribution of injected EVs observed across reviewed studies revealed a similar pattern of tissue targeting, including the liver, spleen, and kidneys, with lower amounts found in the lungs. Moreover, alteration of the surface protein composition, as a result of membrane protein expression in the donor cells, could have caused a significant change in tissue distribution, such as an increase in lung retention of EVs labelled with a CD63-expressed bioluminescent probe when compared to fluorescent or radioactive tracers. Nevertheless, additional studies are needed to elucidate the effects of genetic manipulation of the producing cells on the EV protein composition and the differences in organ targeting. Precise knowledge of EV target tissues, some of which cannot be easily accessed by other treatments, could open the door to more targeted diagnostic and therapeutic strategies.

Novel modalities enhance the sensitivity and detection limit of the employed methods, such as in the case of BRET-based reporters, where the excitation wavelength is shifted towards the red part of the light spectrum, providing deeper tissue penetration and increased sensitivity. Furthermore, the combination of methods such as CT and PET, increases the spatiotemporal information obtained from the EV trafficking. Multimodal imaging, which combines the strengths of various techniques, can offer a better solution to the technical disadvantages of individual imaging modalities.

## Data Availability

No datasets were generated or analysed during the current study.

## Abbreviations

ASCs: Adipose stem cells
AuNPs: Gold nanoparticles
BLI: Bioluminescence imaging
BRET: Bioluminescence resonance energy transfer
CT: Computed tomography
CVP: Caudal vein plexus
DARPin: Designed ankyrin repeat proteins
Gluc: Gaussia princeps luciferase
EVs: Extracellular vesicles
MR: Magnetic resonance
MRI: Magnetic resonance imaging
MSC-exo: EVs derived from mesenchymal stem cells
MVE: Multi-vesicular endosomes
NIR: Near-infrared
NLuc: NanoLuciferase
NOTA: 1,4,7-triazancyclononane-1,4,7-triacetic acid
PEG: Polyethylene glycol
PET: Positron emission tomography
PMN: Pre-metastatic niche
RLuc: Renilla reniformis luciferase
sEVs: Small extracellular vesicles
sgRNA: Single guide RNA
SPECT: Single-photon emission computed tomography
SPIO-His: Superparamagnetic iron oxide particles conjugated with a histidine polypeptide
SPION5: Superparamagnetic iron oxide nanoparticles
TEVs: Tumour-derived extracellular vesicles
YSL: Yolk syncytial layer
